# WHO guidance for digital health: What it means for researchers

**DOI:** 10.1177/2055207619898984

**Published:** 2020-01-08

**Authors:** Tarveen Jandoo

**Affiliations:** Knowledge Management, ZS Associates India, India

**Keywords:** Digital health, guidelines, WHO, reforms, safety

## Abstract

In healthcare, digital solutions have been adopted with zeal, but there is paucity of evidence for benefits and harms of these solutions. The impact, immediate or long term, of digital applications on healthcare has not been assessed. With the overwhelming numbers and types of digital solutions, it is becoming increasingly important to develop evidence-based insights for the integration of these solutions in routine medical care. Digitalization can certainly empower and enable patients and physicians to achieve health objectives. The World Health Organisation has released guidance for digital health after a critical review of available evidence for the benefits, harms, acceptability, feasibility, resource use and equity considerations of digital health interventions. This guidance can potentially inspire and impact future research endeavors for digital applications. In this paper, the guidance has been reviewed in context of the current research situation and insights are shared for researchers engaged in the design and assessment of digital interventions.

## Introduction

Digital applications are increasingly being evaluated in clinical studies.^1^ The United States Food and Drug Administration (USFDA) has identified device software functions qualifying for regulatory evaluation.^2^ The World Health Organisation (WHO) has recently released ‘*Recommendations on digital interventions for health system strengthening*’ to help integration of technology for advancements in healthcare.^3^ With rapid advancements in digital solutions, these guidelines provide useful insights for the greater use of technology. This paper provides an overview of the guidelines and insights for researchers engaged in the development and validation of digital interventions.

The WHO provides a definitive recommendation to adopt digital means for training and education of healthcare professionals such that this can complement, rather than replace, traditional educational endeavors. According to the WHO, this additional delivery channel can help expand access to health education in a more cost-effective manner. However, this intervention should specifically be targeted at continued and in-service training after the required certification in healthcare has been acquired. Although now widely adopted in medicine, digital education measures have been evaluated in heterogenous studies with varying samples and lack of validation of learning content. A systematic review of 93 studies (*N* = 16,895; January 1990 to March 2017) provided empiric evidence for equality of online and blended educational measures to face-to-face trainings for postintervention knowledge, skills, attitude and satisfaction in medical doctors for postregistration training.^4^ In another systematic review of 12 studies (1990–2017), digital education was found to be more effective than traditional education to enhance knowledge and skills for diabetes management.^5^ However, the low quality of these studies makes it difficult to formulate firm conclusions.

The WHO recommends use of mobile devices for birth, death and stock notifications, commodity management, telemedicine, targeted patient communications, health worker decision support and digital tracking of health status and services in specific context and conditions. The WHO has thoroughly evaluated and identified gaps in research for the effectiveness, acceptability, feasibility, requirement of resources and issues around equity and rights for each of these parameters. The WHO recommends any digital health tool or technology to be developed and implemented in accordance with the principles of digital development. There is also guidance for building an enabling environment for fostering the adoption of digital health.

The key implications of the WHO guidelines for research include the following:

### Study designs will evolve

Control and randomization, the most commonly adopted design for clinical trials, has been used for the evaluation of digital applications in healthcare. Examples include trials for a tablet-based app for enhancing participation in informed consent process, a 12-month digital health weight loss intervention for obese patients, and an automated, internet-linked, tablet-based system of monitoring and self-management support in patients with chronic obstructive pulmonary disease.^6–8^ Like in clinical studies for medical devices, challenges like selection bias, learning curve, use of comparator, and generalizability of results may apply to studies for digital applications.^9,10^

With increased options and complexities, digital health will clearly call for innovative trial designs based on the desired outcome measures, targeted populations, technology used, structural levels influenced by digital applications, adaptability and flexibility of digital applications, skills of end-users, and interactions and interdependencies between various digital and non-digital components.^11^ Digital applications may be evaluated in studies designed for value-proposition including controlled before-and-after studies, stepped-wedge randomized controlled trials and interrupted time series studies.^12–16^ There is an evident need for increased knowledge and adoption of the evolving trends in study designs and their impact on regulatory approvals.

### Safety and efficacy are key

The WHO has emphasised the assessment of safety of digital applications, including the possibility of any unintended consequences, as a key gap in research for digital health. Concerns for data privacy and security impact the choice of digital technologies and the design and conduct of studies for these technologies. Digital tools should meet the standards for Health Insurance Portability and Accountability Act of 1996, EU General Data Protection Regulation, and ISO 270001.^17–19^ Data should be pseudonymised and collected, stored, transferred and handled according to applicable local regulations. To comply with the guidance, key strategies for digital health should include safe design, safety reserves, fail-safe and procedural safeguards.^20^

Objective efficacy assessment endpoints can help establish the benefits of digital applications. When compared with the walk test, patient-worn accelerometers have demonstrated sensitivity in detection of deteriorating physical activity with nitrate use.^21^ Mobile devices enable real-world assessment of various parameters such as physical activity, sleep and patient perceptions of health and interventions.^22^

Digital options should ideally be compared with conventional approaches in large-scale well-designed studies. This may be challenging when the chosen conventional outcomes are not the confirmed gold standard. Researchers should analyse and interpret the results without prejudice of implied benefits of digitalization. When no clear benefits are established, researchers and healthcare professionals will need to gracefully embrace the lack of utility of those digital applications and advance to alternate means even if nondigital.

Technology can enable a more ‘real time’ conduct of clinical studies. Though there is no clear directive for the choice of the best technology, careful inclusion of digital applications should be defined when designing a study. Research in digital applications should allow flexibility to accommodate rapid changes rampant in the digital world.

### Acceptability and feasibility should be enhanced

According to the WHO, acceptability of digital health by patients and physicians guides its deployment in research and practice. This synchronizes with patient-centricity in clinical research, where digital health can equip, enable and empower patients for their own health.^23,24^ Development of digital solutions should be focussed on user experience.

Barriers to implementation of digital advances should be identified and addressed. These include the infrastructure, connectivity and quality and validation of the digital applications. Healthcare ecosystems in developing countries may not be mature enough to adopt and integrate digital tools for healthcare. According to the WHO, ‘frameworks such as RE-AIM (Reach, Effectiveness, Adoption, Implementation, and Maintenance) may be useful in structuring the implementation research.’

Legal, ethical and regulatory milieu should be strengthened to allow the easy adoption of technology in healthcare. The USFDA has formulated a Digital Health Innovation Action Plan to ensure timely access to high-quality, safe and effective digital health products.^25^ The Electronic Patient-Reported Outcome Consortium has developed recommendations for the selection and evaluation of wearable technology applications in clinical trials.^26^ The Clinical Trials Transformation Initiative has also released recommendations for advancing the use of mobile technologies for data capture and improved clinical trials.^27^

### Knowledge, attitude and behaviours should be measured

Some of the key gaps described by the WHO are in the knowledge and attitude of patients and healthcare professionals and their behaviours towards digital health. These are primarily for interventions like targeted patient communications, health worker decision support, digital tracking and mobile learning. Satisfaction levels, apprehensions, hesitations and fears should be identified, and measures should be defined for mitigation of the same. The knowledge–attitude–behaviour approach has been used extensively to study the impact of education on patient and provider acceptance as well as understanding of healthcare measures and practices and associated safety.^28–31^ More of these endeavours will help to plan dissemination and enable adoption of digital health.

### Cost-effectiveness will add value

Digital interventions can save time and effort, and translate into potential savings. The lack of assessments for cost-savings is widely recognized. In a recent systematic literature review of online digital education for the postregistration training of medical doctors, the authors identified no studies reporting cost-effectiveness among the studies that compared online distance education or blended education with self-directed/face-to-face learning.^4^ The WHO recommends assessments of long-term costs with ‘accounting of amortization and maintenance of equipment and the continuous user support required.’

Reforms are the next immediate step. Researchers should not neglect the guidance for enabling access to healthcare. The WHO recommends the use of stock notification and commodity management via mobile devices. However, this applies to settings where supply chain management systems are enabled to adopt, handle and respond to such notifications. This is likely to ensure the availability of medical commodities with better management of logistics. This calls for awareness, skills, infrastructure, financing, and systems and regulations for strengthening the supply chain management. An example is the Mega Drug Distribution Centres and National Drug Distribution Guidelines to improve access to quality medicines in Nigeria.^32^ Such guidelines will need to be updated to include specifications for mobile devices.

In summary, there is a need for evidence-based digital health interventions, measurements of which in clinical trials can support regulatory assessments and decisions and labelling claims.^26^ Besides a revolution and an evolution, digital health is a cultural reform in healthcare.^33^ With the proliferation of a plethora of digital tools, researchers need to avoid an overenthusiastic approach that will introduce prejudice and inequities. Disparities in digital health are a common challenge and should be addressed during development to enable uniform and universal benefits to the end users. Sustained efforts should be made to develop novel digital solutions that are applicable and acceptable in routine patient care in real life.
